# The association between chronic disease resource utilization and illness uncertainty in COPD patients: a latent profile analysis

**DOI:** 10.7717/peerj.20674

**Published:** 2026-01-26

**Authors:** Yangjuan Bao, Lili Yang, Jing-yi Zhao, Zhiqian Wang, Leimian Fu, Min Fang, Jin’e Lin

**Affiliations:** 1 Department of Nursing, The Fourth Affiliated Hospital of School of Medicine, and International School of Medicine, International Institutes of Medicine, Zhejiang University, Yiwu, Zhejiang, China; 2 Department of Nursing, Sir Run Run Shaw Hospital, Zhejiang University School of Medicine, Hangzhou, Zhejiang, China; 3 Affiliated Hangzhou First People’s Hospital, School of Medicine, Westlake University, Hangzhou, Zhejiang, China; 4 Yiwu Second People’s Hospital, Yiwu, Zhejiang, China

**Keywords:** COPD, Chronic disease resource utilization, Illness uncertainty, Latent profile analysis

## Abstract

**Objective:**

This study aimed to identify distinct patterns of chronic disease resource utilization among patients with chronic obstructive pulmonary disease (COPD) and to examine their association with illness uncertainty.

**Design:**

A cross-sectional study.

**Methods:**

This study enrolled COPD patients hospitalized in the Department of Respiratory Medicine at a tertiary hospital in Zhejiang Province, China, between April and December 2023. All participants completed a general information form, the Chronic Illness Resource Survey (CIRS), and the Mishel Uncertainty in Illness Scale (MUIS). Latent profile analysis (LPA) was conducted to identify subgroups of resource utilization patterns. Subsequently, hierarchical linear regression was employed to assess the associations between these patterns and illness uncertainty. Ethical approval was obtained from the Institutional Review Board of the Fourth Affiliated Hospital of Zhejiang University (Approval No. K2022057).

**Results:**

A total of 308 participants were included. Two latent classes of resource utilization were identified: the Suboptimal Utilization Group (*n* = 209) and the Effective Utilization Group (*n* = 99). Patients in the effective utilization group reported significantly lower levels of illness uncertainty (R^2^ = 0.587, *p* < 0.001).

**Conclusions:**

Distinct patterns of chronic disease resource utilization exist among COPD patients and are significantly associated with illness uncertainty. Healthcare providers should recognize these subgroups and implement targeted interventions to enhance access to disease-related support resources, thereby mitigating illness uncertainty.

**Implications:**

Understanding COPD patients’ varying patterns of resource utilization enables healthcare professionals and related industries to deliver personalized, resource-based interventions tailored to individual needs, ultimately reducing illness-related uncertainty and improving disease management outcomes.

## Introduction

Chronic obstructive pulmonary disease (COPD) is a heterogeneous pulmonary disorder characterized by persistent respiratory symptoms and progressive airflow limitation resulting from airway and/or alveolar abnormalities (Celli et al., 2022). As a global public health challenge, COPD poses significant threats to human health due to its high morbidity and mortality rates (GBD 2021 Causes of Death Collaborators, 2024; Naeem et al., 2025). In 2021, China recorded 50.6 million prevalent COPD cases, 4.4 million incident cases, 1.29 million deaths, and 23.6 million disability-adjusted life years (Liu et al., 2025). Furthermore, COPD contributes substantially to excessive healthcare resource utilization and expenditure worldwide, with particularly severe impacts in developing countries. A macroeconomic modeling study covering 204 countries and territories projected the COPD-related economic burden from 2020 to 2050, revealing that China is expected to experience the greatest financial losses, estimated at 120 billion RMB (Chen et al., 2023). Concurrently, COPD imposes a significant symptom burden, where recurrent exacerbations lead to frequent hospitalizations that severely impair patients’ work capacity and quality of life; this creates substantial caregiving burdens for families, thereby intensifying both individual and global disease burdens (Zhang et al., 2025).

COPD is characterized by irreversible pulmonary dysfunction associated with a high disability burden and life-threatening complications. The recurrent nature of disease exacerbations generates considerable uncertainty regarding treatment effectiveness and disease management, while unpredictable disease progression and outcomes further heighten patients’ illness uncertainty (Hanania & O’Donnell, 2019). This uncertainty primarily stems from patients’ limited disease-related knowledge, which impairs their understanding of disease processes and prognostic expectations (Mishel, 1988). Such psychological distress exacerbates anxiety, depression, and emotional distress (Cui, Li & Wang, 2021; Kassem et al., 2019), adversely affecting treatment adherence, quality of life, and health outcomes (Roberts et al., 2020). Notably, these negative emotional states may create a vicious cycle by compromising patients’ engagement in clinical management and potentially leading to treatment discontinuation (Wang et al., 2022). Comparative research has shown that illness uncertainty levels in COPD patients are approximately twice as high as those observed in non-COPD populations (Hanania & O’Donnell, 2019). These findings underscore the urgent need to explore the mechanisms underlying illness uncertainty in COPD to inform the development of effective coping interventions that alleviate disease-related psychological and behavioral burdens.

Research has established a link between resource utilization and illness uncertainty in chronic conditions (Lee, Lee & Kwon, 2023). Effective use of resources enhances patients’ understanding of their illness and strengthens their coping capacity, thereby reducing uncertainty (Alshraifeen et al., 2020). However, the effectiveness of resources depends on their quality and contextual fit; inappropriate or mismatched support may even exacerbate distress (Hoth et al., 2015; Zhang et al., 2024). In addition, most existing studies have examined isolated resources, such as family or healthcare providers, while overlooking the multidimensional and interrelated nature of resource utilization across intrapersonal, interpersonal, environmental, and cultural levels (Durna, Durna & Seçer, 2022; Kim, Yeom & Jeon, 2020).

The Chronic Illness Resource Survey (CIRS) (Glasgow et al., 2000), grounded in socio-ecological theory, provides a comprehensive framework for evaluating patients’ use of diverse resources across these four hierarchical levels. However, whether distinct patterns of resource utilization exist among patients with COPD and how these patterns relate to illness uncertainty remain unexplored. Conventional variable-centered approaches that rely on cumulative scale scores fail to capture heterogeneity among patients, as they overlook influential demographic and contextual factors—such as education, economic stress, and age—that shape how resources are accessed and used.

To address these gaps, the present study employs latent profile analysis (LPA), a person-centered statistical approach, to identify homogeneous subgroups of COPD patients based on multidimensional resource utilization patterns measured by the CIRS (Hao et al., 2025). This method enables the incorporation of key covariates to validate the distinct profiles and to describe their sociodemographic and psychosocial characteristics. The study aims to (1) identify distinct latent classes of resource utilization among COPD patients, and (2) examine the associations between these resource utilization patterns and illness uncertainty. The findings are expected to provide a theoretical foundation for developing precision interventions tailored to the specific resource configurations and uncertainty experiences of different patient subgroups.

## Materials and Methods

The study adhered strictly to STROBE (Strengthening the Reporting of Observational Studies in Epidemiology) guidelines for cross-sectional research to ensure methodological rigor and reporting completeness.

### Study design and procedures

We conducted a cross-sectional survey at a tertiary Grade A hospital in Zhejiang Province, China, between April and December 2023. Prior to data collection, the principal investigator trained two research assistants in standardized procedures for administering the questionnaire, including distribution, completion instructions, and the use of uniform guidance. After obtaining informed consent, the questionnaires were administered in person with brief instructions on how to complete them. For participants who were unable to complete the questionnaire independently, the research assistants conducted face-to-face interviews, reading each item *verbatim* and recording the participants’ responses. Each questionnaire required approximately 10 min to complete. All questionnaires were collected and checked on site immediately after completion, and any ambiguities were clarified in real time to ensure data completeness. Of the 320 questionnaires distributed, 308 valid responses were obtained, yielding a 94.4% valid response rate.

### Participants

Participants were recruited through convenience sampling from patients with COPD who were hospitalized in the respiratory intensive care unit of a tertiary hospital in Zhejiang Province, China. Inclusion criteria were as follows: (1) diagnosis of COPD according to the Global Initiative for Chronic Obstructive Lung Disease (2023) guidelines, with a postbronchodilator ratio of the forced expiratory volume in one second (FEV1) to the forced vital capacity (FVC) < 0.70 (Agustí et al., 2023); (2) disease duration ≥6 months; (3) age ≥18 years; (4) clear consciousness and ability to communicate; and (5) voluntary participation with signed informed consent. Exclusion criteria included: (1) severe comorbidities or organ dysfunction (*e.g*., cardiovascular/cerebrovascular diseases, hepatic/renal dysfunction, or malignancies); (2) physical disabilities, psychiatric disorders, or neurological diseases; (3) other pulmonary diseases such as asthma or tuberculosis; and (4) critical illness requiring non-invasive or invasive mechanical ventilation. According to Hsieh, Bloch & Larsen (1998), the sample size for logistic regression should be 5–10 times the number of independent variables. With 14 predictors, at least 140 participants were required; allowing for a 10% attrition rate, the final target sample size was set at 155. For the LPA, a minimum of 200 participants was considered sufficient to ensure model stability (Wang & Wang, 2019).

### Measures

#### Demographic and clinical characteristics questionnaire

Based on previous literature (Oostrik et al., 2023), we developed a comprehensive demographic questionnaire for COPD patients to collect information on: (1) basic characteristics, such as gender, age, educational level, and marital status; (2) residential information, including location (urban/rural) and living arrangements; (3) socioeconomic status indicated by monthly household income *per capita* and economic pressure; (4) clinical characteristics, including smoking status, duration of COPD, pulmonary function classification according to GOLD criteria (Grade I: FEV_1_ ≥80% predicted; Grade II: FEV_1_ 50–79% predicted; Grade III: FEV_1_ 30–49% predicted; Grade IV: FEV_1_ <30% predicted), comorbidities, and the number of COPD-related hospitalizations in the past 12 months.

#### Chronic Illness Resource Survey (CIRS)

The Chronic Illness Resource Survey (CIRS) was originally developed by Glasgow et al. (2000) and later cross-culturally adapted for type 2 diabetes patients by Zhong et al. (2014). To ensure its suitability for COPD patients, our study performed targeted modifications and comprehensive validation of this 16-item instrument (Bao et al., 2025). The questionnaire assesses five dimensions: (1) healthcare team support (three items), (2) family/friend support (three items), (3) individual coping strategies (three items), (4) neighborhood/community resources (four items), and (5) media/policy resources (three items). Each item is rated on a 5-point Likert scale from 1 (“never”) to 5 (“very often”). The total score is calculated as the sum of all item scores divided by the number of items (range: 1–5), with scores ≥3 indicating adequate resource utilization, while scores <3 suggest suboptimal utilization. In our study, the questionnaire demonstrated good reliability, with an overall Cronbach’s α of 0.819 and subscale coefficients ranging from 0.803 to 0.891.

#### Mishel Uncertainty in Illness Scale (MUIS)

The Mishel Uncertainty in Illness Scale (MUIS) was originally developed by an American nursing expert (Mishel, 1988) and has been widely used to assess illness uncertainty among hospitalized adults. It was later translated into Chinese by Xu & Huang (1996), who tested its reliability and validity, yielding a 25-item Chinese version. This adapted scale comprises two dimensions: ambiguity (15 items) and complexity (10 items). Each item is rated on a 5-point Likert scale from 1 (“strongly disagree”) to 5 (“strongly agree”), with total scores ranging from 25 to 125, where higher scores indicate greater illness-related uncertainty (Hoth et al., 2015). Previous studies reported good reliability, with a Cronbach’s α of 0.864, and in our study, the scale demonstrated similarly strong internal consistency (Cronbach’s α = 0.840).

#### Ethical considerations

This study received ethical approval from the Institutional Review Board of the Fourth Affiliated Hospital of Zhejiang University (Approval No. K2022057) prior to data collection. All participants provided written informed consent by the principles outlined in the Declaration of Helsinki.

#### Statistical analysis

The analysis consisted of two parts. The first part involved a LPA based on the five dimensions of chronic disease resource utilization. An unconditional model was employed, incorporating all dimension scores from the CIRS as observed variables, to identify latent classes representing distinct patterns of resource utilization at the individual level.

The determination of the optimal number of latent classes was based on model fit indices. Lower values of the Akaike Information Criterion (AIC), Bayesian Information Criterion (BIC), and adjusted BIC (aBIC) indicated better model fit. Comparative assessment using the Bootstrapped Likelihood Ratio Test (BLRT) and the Lo-Mendell-Rubin adjusted likelihood ratio test (LMR) demonstrated that a k-class model provided superior fit to a (k − 1)-class model when the following criteria were met: lower AIC, BIC, and aBIC values, along with statistically significant BLRT and LMR results (*p* < 0.05) (Kim, 2014; Adhikari, Devkota & Cesuroglu, 2021). An entropy value >0.8 indicated a classification accuracy exceeding 90% (Wang et al., 2017). The final determination of class numbers incorporated both statistical indicators and clinical interpretability.

Categorical variables were summarized as frequencies and percentages, with intergroup comparisons performed using chi-square tests. The normality of continuous variables was assessed using the Shapiro–Wilk test and Q-Q plots. Normally distributed continuous variables are expressed as means ± standard deviations, and group comparisons were conducted using t-tests or analysis of variance (ANOVA). For comparisons involving unequal variances, Welch’s t-test was applied. *Post-hoc* analyses were performed using Bonferroni or Games–Howell tests, as appropriate.

In the second phase, hierarchical linear regression analysis was performed to first identify sociodemographic factors associated with illness uncertainty, and second, to evaluate the relationships between latent classes of chronic disease resource utilization and illness uncertainty. Illness uncertainty was treated as the dependent variable, while statistically significant sociodemographic factors and resource utilization patterns were included as independent variables.

## Results

### Demographic characteristics of participants

A total of 320 questionnaires were distributed, and 308 valid responses were ultimately included in the analysis (Table 1). The mean age of patients was 72.16 ± 9.75 years. Among them, 93.2% were male, 71.1% had an education level of primary school or below, 76.0% resided in rural areas, and 59.1% had a monthly income of less than 5,000 RMB.

**Table 1:** Baseline sociodemographic and clinical characteristics of the total sample.

Variables		Total sample (*n* = 308)	%
Gender			
Male		287	93.2
Female		21	6.8
Age, years			
<65		41	13.3
65–74		147	47.7
75–84		82	26.6
≥85		38	12.3
Marital status			
Single		3	1.0
Married		235	76.3
Divorced		3	1.0
Widowed		67	21.8
Residence			
Urban		74	24.0
Rural		234	76.0
Living status			
Alone		21	6.8
Couple		209	67.9
With children or relatives		65	21.1
Senior care institution		13	4.2
Education level			
Primary school or below		219	71.1
Junior high school		53	17.2
High school or above		36	11.7
Average monthly income (RMB)			
≤5,000		182	59.1
>5,000		126	40.9
Economic pressure			
No		240	77.9
Yes		68	22.1
COPD grade			
Grade 1		21	6.8
Grade 2		64	20.8
Grade 3		120	39.0
Grade 4		103	33.4
Smoking status			
Never		78	25.3
Current		55	17.9
Former		175	56.8
Disease duration, years			
1–5		56	18.2
6–10		112	36.4
>10		140	45.5
Hospital visits (Last Year)			
0–1		168	54.5
2–3		117	38.0
≥4		23	7.5
Number of comorbidities			
0–2		117	38.0
≥3		191	62.0

### Latent profiles of chronic disease resource utilization

LPA was conducted based on the five dimensions of chronic disease resource utilization. Models with one to five latent classes were fitted (Table 2). The 4- and 5-class models were excluded because one class in each model contained less than 5% of the sample, which is considered unstable for interpretation. The two-class model showed higher entropy and better theoretical interpretability than the three-class model and was therefore selected as the optimal solution. Classification accuracy was high, with average posterior probabilities of 97.4% for Class 1 and 94.2% for Class 2, confirming the reliability of the two-class solution.

**Table 2:** Goodness-of-fit indices for latent profile analysis of chronic disease resource utilization patterns in COPD patients.

Model	AIC	BIC	aBIC	Entropy	LMRT	BLRT	Group size for each class
1	2,533.667	2,570.968	2,539.252	–	–	–	308
2	2,023.054	2,082.736	2,031.991	0.877	0.0000	0.0000	209/99
3	1,939.784	2,021.846	1,952.071	0.860	0.0578	0.0000	180/36/92
4	1,875.508	1,979.951	1,891.147	0.894	0.0107	0.0000	6/36/175/91
5	1,827.846	1,954.669	1,846.836	0.912	0.1022	0.0000	6/85/39/4/174

**Figure 1:**
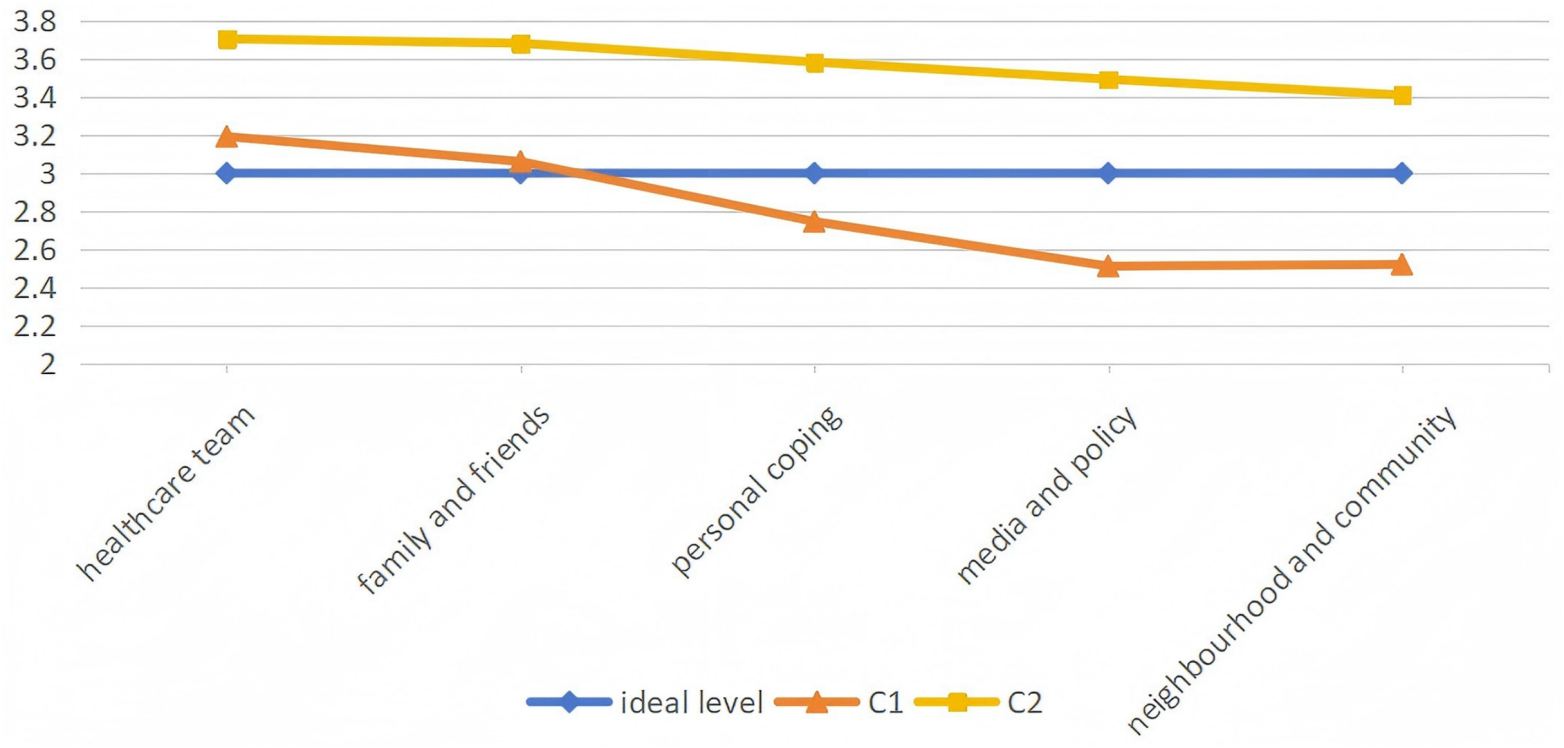
Two latent profiles of chronic disease resource utilization in COPD patients; mean domain score by chronic illness resources latent profile.

Figure 1 presents the dimensional scores of the two COPD patient subgroups. According to the questionnaire’s scoring system, optimal chronic disease resource utilization corresponds to a score of 3 across all five dimensions (represented by the “optimal level” line in Fig. 1). Group C1 scored significantly below the optimal level (*p* < 0.05) in three dimensions, although scores for healthcare team (3.192) and family/friend support (3.061) were adequate. This overall pattern defined Group C1 as the “Suboptimal Utilization Group.” In contrast, Group C2 consistently achieved optimal scores across all dimensions, which was therefore classified as the “Effective Utilization Group.” Distribution analysis indicated that 67.9% (*n* = 209) of participants belonged to the Suboptimal Utilization Group, while 32.1% (*n* = 99) comprised the Effective Utilization Group.

### Comparative analysis of COPD patient characteristics between latent classes

Using the two latent profiles of chronic disease resource utilization identified in COPD patients as grouping variables, we conducted univariate analyses to examine their associations with sociodemographic and clinical characteristics. Chi-square tests were employed to assess differences between the two subgroups. The results demonstrated that gender (*p* = 0.021), age (*p* = 0.002), educational level (*p* < 0.001), average monthly income (*p* = 0.018), economic pressure (*p* < 0.001), smoking status (*p* = 0.001), number of hospital visits in last year (*p* < 0.001), and comorbidities (*p* < 0.001) were significantly associated with chronic disease resource utilization patterns (Table 3). Specifically, compared to the C1 group (Suboptimal Utilization), the C2 group (Effective Utilization) consisted of younger patients with fewer comorbidities, higher education levels, higher household income, lower financial stress, and fewer acute exacerbation-related hospitalizations.

**Table 3:** Comparison of sociodemographic and clinical characteristics between the two identified profiles.

Variables		C1 (*n* = 209, %)	C2 (*n* = 99, %)	χ^2^	*p*
Gender					
Male		190 (90.9)	97 (98.0)	5.286	0.021
Female		19 (9.1)	2 (2.0)		
Age, years					
<65		28 (13.4)	13 (13.1)	14.965	0.002
65–74		85 (40.7)	62 (62.6)		
75–84		65 (31.1)	17 (17.2)		
≥85		31 (14.8)	7 (7.1)		
Marital status					
Single		2 (1.0)	1 (1.0)	2.110	0.577
Married		162 (77.5)	73 (73.7)		
Divorced		3 (1.4)	0 (0.0)		
Widowed		42 (20.1)	25 (25.3)		
Residence					
Urban		43 (20.6)	31 (31.3)	4.244	0.055
Rural		166 (79.4)	68 (68.7)		
Living status					
Alone		12 (5.7)	9 (9.1)	4.797	0.187
Couple		140 (67.0)	69 (69.7)		
With children or relatives		45 (21.5)	20 (20.2)		
Senior care institution		12 (5.7)	1 (1.0)		
Education level					
Primary school or below		168 (80.4)	51 (51.5)	27.320	<0.001
Junior high school		25 (12.0)	28 (28.3)		
High school or above		16 (7.7)	20 (20.2)		
Average monthly income (RMB)					
≤5,000		133 (63.6)	49 (49.5)	5.557	0.018
>5,000		76 (36.4)	48 (48.5)		
Economic pressure					
No		149 (71.3)	91 (91.9)	16.615	<0.001
Yes		60 (28.7)	8 (8.1)		
COPD grade					
Grade 1		11 (5.3)	10 (10.1)	7.065	0.070
Grade 2		40 (19.1)	24 (24.2)		
Grade 3		79 (37.8)	41 (41.4)		
Grade 4		79 (37.8)	24 (24.2)		
Smoking status					
Never		57 (27.3)	21 (21.2)	13.036	0.001
Current		26 (12.4)	29 (29.3)		
Former		126 (60.3)	49 (49.5)		
Disease duration, years					
1–5		41 (19.6)	15 (15.2)	0.909	0.417
6–10		75 (35.9)	37 (37.4)		
>10		93 (44.5)	47 (47.5)		
Hospital visits (Last year)					
0–1		92 (44.0)	76 (76.8)	29.203	<0.001
2–3		97 (46.4)	20 (20.2)		
≥4		20 (9.6)	3 (3.0)		
Number of comorbidities					
0–2		65 (31.1)	52 (52.5)	13.09	<0.001
≥3		114 (68.9)	47 (47.5)		

### Differences in illness uncertainty between latent classes of COPD patients

We further examined differences in illness uncertainty between the two latent classes and explored associated factors. Statistically significant differences were observed in both the dimensional and total MUIS scores between the two groups. Specifically, the C1 group exhibited significantly higher scores than the C2 group for the complexity dimension (*p* < 0.001), the ambiguity dimension (*p* < 0.001), and the total MUIS score (*p* < 0.001) (Table 4).

**Table 4:** Illness uncertainty scores by resource utilization profile.

Variables	C1	C2	*T*	*p*
Complexity dimension	31.74 ± 2.05	27.08 ± 2.78	14.863	<0.001
Ambiguity dimension	48.87 ± 5.05	39.64 ± 5.23	14.804	<0.001
Total MUIS score	80.61 ± 6.05	66.72 ± 7.45	16.205	<0.001

**Note:**

C1, Suboptimal utilization; C2, effective utilization.

Initial univariate linear regression analysis identified several demographic and clinical variables significantly associated with illness uncertainty (all *p* < 0.05), including age, residence, education level, monthly household income, financial stress, pulmonary function grade, smoking status, frequency of acute exacerbation-related hospitalizations in the past year, and number of comorbidities. Subsequently, hierarchical linear regression was conducted with illness uncertainty as the dependent variable, including the significant demographic variables and latent profiles of chronic disease resource utilization as independent variables. The results demonstrated a significant association between patterns of chronic disease resource utilization and illness uncertainty in patients with COPD (R^2^ = 0.587, *p* < 0.001). Overall, patients in the C2 group exhibited consistently lower levels of illness uncertainty compared to those in the C1 group (Table 5).

**Table 5:** Association between profiles of chronic illness resource utilization and illness uncertainty in linear regression analysis.

Variables	Beta	*t*	*p*
Constant		22.727	<0.001
Chronic illness resource utilization			
C1 group	0.620	13.236	<0.001
Age, years			
<65	−0.120	−1.914	0.057
65–74	−0.164	−2.338	0.020
75–84	−0.060	−1.009	0.314
Residence			
Urban	−0.123	−2.458	0.015
Education level			
Primary school or below	−0.088	−1.244	0.214
Junior high school	−0.093	−1.517	0.130
Average monthly income (RMB)			
≤5,000	0.016	0.272	0.786
Economic pressure			
No	−0.061	−1.327	0.186
COPD grade			
Grade 1	−0.127	−2.444	0.015
Grade 2	0.062	1.228	0.220
Grade 3	0.028	0.584	0.559
Smoking status			
Never	0.050	1.145	0.253
Current	0.023	0.487	0.627
Hospital visits (last year)			
0–1	−0.252	−2.992	0.003
2–3	−0.172	−2.190	0.029
Number of comorbidities			
0–2	0.028	0.593	0.554
F	18.424		<0.001
R^2^	0.587		

## Discussion

The present study revealed two distinct patterns of chronic disease resource utilization among COPD patients, which were significantly associated with their demographic and clinical characteristics. Specifically, factors such as age ≥85 years, rural residence, GOLD grade 4, and ≥4 acute exacerbation hospitalizations in the past year were all linked to higher levels of illness uncertainty. Consequently, the level of resource utilization emerged as a significant factor, with patients in the “effective utilization group” exhibiting significantly lower illness uncertainty than those in the “suboptimal utilization group.”

Among COPD patients, chronic disease resource utilization can be classified into two distinct patterns: C1 (Suboptimal utilization group) and C2 (effective utilization group). The C1 pattern was predominant, comprising 67.9% of participants, whereas only 32.1% belonged to the C2 group, which signifies effective resource utilization for self-management. This distribution is consistent with prior research (Liu et al., 2024). A detailed comparison revealed that while the C1 group reached optimal levels only in the healthcare team and family/friends dimensions, it scored below optimal in personal coping, media/policy, and neighborhood/community—with the media/policy dimension being the lowest. In contrast, the C2 group achieved optimal levels across all five dimensions, although the neighborhood/community dimension remained the lowest within this group.

In both patient groups, the healthcare team dimension received the most optimal scores, a finding consistent with multiple domestic and international studies (Liu et al., 2024; Heimel et al., 2021). This underscores the universally crucial role healthcare professionals play as a support resource in COPD management. In the Chinese context, this high reliance may be partly attributed to significant advancements in the healthcare system, including continuously elevated recruitment standards and improved overall professional competence of medical staff. Under the prevailing healthcare model, the professional informational support from medical staff is highly recognized by patients and acts as a guiding force within their resource networks (Watson & Wilkinson, 2022), leading to greater dependence on healthcare professionals for disease control and decision-making (Le et al., 2023). The patient-provider connection is further strengthened by the rapid development of “Internet+ Healthcare”. Emerging service models like Internet hospitals have broken temporal and spatial barriers to medical resources, significantly enhancing accessibility (Zhao et al., 2024). This effective integration of online and offline services facilitates closer and more sustained connections between patients and healthcare providers. These findings carry important implications for clinical practice: On one hand, it remains essential to strengthen the professional development of medical teams to maintain and improve service quality; on the other hand, further exploration of innovative “Internet + Healthcare” models should be pursued to optimize resource allocation and establish a more comprehensive and convenient medical support system for COPD patients.

This study revealed that in the C1 group, the media and policy dimension scored the lowest, which was significantly lower than the findings reported by Wang et al. (2024) in stroke patients. This discrepancy may be attributed to varying levels of public awareness and policy support across different chronic conditions. Despite recent nationwide efforts to standardize COPD management in primary care settings, societal attention to COPD remains inadequate compared to other chronic diseases like diabetes and hypertension. The underutilization of media resources is particularly evident when viewed against the global shift towards digital health management. For instance, Heimel et al. (2021) demonstrated in their German and Swiss cohorts that approximately 75% of COPD patients sought disease-related information online, with about 33% utilizing social media platforms for peer communication—reflecting a global trend toward digital health management. In the C1 cohort of this study, 45.9% of patients were aged over 75 or older. Due to limited digital literacy, the majority of elderly patients demonstrated difficulty in proficiently utilizing computers or smartphones to access reliable disease-related information. Additionally, some patients reported challenges in distinguishing credible online information and filtering effective content. Although specialized medical applications currently provide health management services, the utilization rate among the elderly population remains low. Existing health education resources predominantly target younger demographics (Zhou, Bai & Wang, 2024). Consequently, there is an urgent need to optimize age-friendly designs in digital health services and reduce accessibility barriers to improve health information acquisition among middle-aged and elderly patients.

Our study demonstrated significantly higher illness uncertainty in elderly COPD patients. This phenomenon may be attributed to three main factors: first, age-related progressive decline in physical function and cognitive capacity; second, increased complexity of disease management due to multiple comorbidities; third, reduced ability to comprehend complex medical information. These factors collectively contribute to heightened anxiety regarding disease progression and mortality risk (Xie et al., 2022). Michalovic et al. (2020) further confirmed that elderly COPD patients tend to adopt less effective coping strategies and experience greater psychological distress. Furthermore, illness uncertainty was compounded by socioeconomic factors, particularly for rural residents. The uneven distribution of medical resources is particularly pronounced in rural areas (Wu et al., 2024), which are characterized by insufficient specialist care, inadequate coverage of pulmonary rehabilitation programs, and low health literacy among residents (Li, Zhang & Jian, 2023). Our findings support this reality, revealing that rural patients frequently experience delayed diagnosis and suboptimal disease management due to poor healthcare accessibility. These barriers exacerbate their sense of helplessness and uncertainty regarding disease control—a conclusion consistent with the significant geographical disparities in COPD outcomes highlighted by Naeem et al. (2025).

Patients with GOLD grade 4 demonstrated significant clinical differences compared to those with GOLD grade 1. Severe airflow limitation resulted in markedly impaired physical function and increased dependence on daily activities while simultaneously triggering fears of rapid disease progression. The irreversible nature of advanced COPD exacerbated patients’ survival concerns, forcing them to confront the chronic progression and poor prognosis of their disease (Henoch et al., 2021). The GOLD Report (Patel, 2024) indicates that very severe COPD patients, due to heavy symptom burden, uncertain prognosis, and frequent acute exacerbations, are particularly prone to developing anxiety and depressive symptoms. This study further confirmed a significant positive correlation between the frequency of acute exacerbations and illness uncertainty. The impact of exacerbations is substantial; for instance, a systematic review by Ruan et al. (2023) revealed a one-year readmission rate as high as 37% following acute exacerbations. Given the chronic and progressive nature of COPD, patients may experience frequent hospitalizations due to increasing exacerbation episodes. When patients undergo multiple disease-related hospitalizations within a year, this indicates more frequent disease recurrence and clinical instability. Repeated hospitalizations not only impose substantial financial burdens and negative psychological impacts on patients but also undermine their confidence in treatment efficacy and disease prognosis (Jørgensen et al., 2021). Furthermore, the recurrent nature of symptom fluctuations further limits patients’ ability to engage socially. Lacking an adequate understanding of their disease progression, patients experience heightened uncertainty regarding their clinical trajectory, thereby exacerbating the uncertainty of their illness.

Based on these research findings, we recommend that future studies focus on the following directions: First, in-depth investigation of the specific mechanisms through which social determinants (*e.g*., social support and healthcare accessibility) influence illness uncertainty. Second, optimizing targeted intervention strategies for different patient populations includes implementing multidisciplinary team management for elderly patients, simplifying health education materials, and strengthening family support systems. For rural patients, digital solutions should be developed, such as remote monitoring and short video health education, to improve access to health information. For individuals who frequently experience exacerbations, establishing psychological support pathways is recommended. In clinical practice, healthcare providers should emphasize personalized communication strategies to enhance patients’ understanding of disease progression, thereby effectively reducing uncertainty and ultimately improving quality of life.

This study represents the first investigation into the relationship between latent characteristics of chronic disease resource utilization and illness uncertainty in COPD patients, providing insights for implementing targeted interventions. Our findings demonstrate that COPD patients’ utilization of chronic disease resources can be categorized into two distinct clusters: the majority primarily utilize disease-related resources provided by medical teams, while underutilizing other available resources. These two patient groups exhibited significant differences in demographic characteristics including age, residential location, and pulmonary function grade. These results enable healthcare institutions to identify patterns and characteristics of chronic disease resource utilization among different COPD patient populations while highlighting the role of optimized resource utilization in reducing illness uncertainty, thereby offering novel perspectives for improving patients’ quality of life.

### Limitations

This study has several limitations. First, both the utilization of chronic disease resources and the scores of illness uncertainty were self-reported by patients, which may introduce potential reporting bias. Second, the sample consisted of patients from a single tertiary hospital, which led to limitations in sample selection. Future research should involve larger-scale, multi-regional, and multi-level hospital investigations to enhance sample diversity.

## Conclusions

Enhanced attention should be directed toward chronic disease resource utilization among patients with COPD. In this study, we classified COPD patients into two distinct latent categories based on their patterns of resource utilization. The inverse relationship between resource utilization and illness uncertainty underscores that enhancing patients’ access to and use of multidimensional support resources could be a pivotal strategy in mitigating the psychological burden of uncertainty in COPD management. These findings may provide valuable insights for healthcare providers to (1) identify different COPD patient subtypes, (2) differentiate between patients with optimal *vs* suboptimal resource utilization, and (3) implement tailored interventions to promote better resource utilization, thereby potentially reducing illness-related uncertainty in this population.

## Supplemental Information

10.7717/peerj.20674/supp-1 Supplemental Information 1 Raw data.

10.7717/peerj.20674/supp-2 Supplemental Information 2 Codebook for Raw Data.

## Additional Information and Declarations

### Data Availability

The following information was supplied regarding data availability:

The raw data is available in the Supplemental File.
