# Supplementary material for: The association between chronic disease resource utilization and illness uncertainty in COPD patients: a latent profile analysis
Source: PeerJ. 2026 Jan 26;14:e20674. doi: 10.7717/peerj.20674 (PMC12854130; doi:10.7717/peerj.20674)
Supplement: Supplemental Information 2 [file peerj-14-20674-s002.docx]

The non-English text "基本资料" in the file "raw_date11.18.xlsx" has been translated into English as "Basic Information".
